# Genome-wide CRISPR screen identifies GNE as a key host factor that promotes influenza A virus adsorption and endocytosis

**DOI:** 10.1128/spectrum.01643-23

**Published:** 2023-11-15

**Authors:** Tianxin Ma, Shiqi Niu, Zihua Wu, Shenghui Pan, Chenyang Wang, Xiaona Shi, Minghao Yan, Bangfeng Xu, Xingpo Liu, Luzhao Li, Dawei Yan, Qiaoyang Teng, Chunxiu Yuan, Xue Pan, Zhifei Zhang, Hoang Minh Duc, Zejun Li, Qinfang Liu

**Affiliations:** 1 Shanghai Veterinary Research Institute, Chinese Academy of Agricultural Sciences, Shanghai, China; 2 Faculty of Veterinary Medicine, Vietnam National University of Agriculture, Hanoi, Vietnam; Shandong First Medical University, Jinan, Shandong, China

**Keywords:** influenza A virus, GNE, sialic acid, adsorption

## Abstract

Replication of influenza A virus (IAV) is highly reliant on host cell function, and to identify the key host factor required in the influenza A virus life cycle, a genome-wide CRISPR/Cas9 knockout (KO) screen was conducted in A549 cells infected by H1N1 influenza virus. The results showed that glucosamine (UDP-N-acetyl)-2-epimerase/N-acetylmannosamine kinase (GNE) plays a crucial role in the replication of influenza A virus, and knockout of the GNE significantly reduced the replication of multiple subtype influenza A viruses in A549 cells, and restoration of the GNE expression in the GNE-knockout cells partially recovered IAV infection. Additionally, overexpression of the GNE in wild-type (WT) cells promoted IAV infection to a certain extent. Further study showed that the GNE is involved in α-2,3- and α-2,6-linked sialic acid (Sia) synthesis, which is the major receptor of the influenza A virus, and the GNE knockout downregulated the α-2,3- and α-2,6-linked sialic acid expression. In summary, the knockout of the GNE inhibits adsorption and endocytosis of IAV. This study provides a new approach to elucidate the replication mechanism of IAV and identifies GNE as a potential target for anti-influenza drug development.

**IMPORTANCE:**

Influenza A virus infection requires the assistance of the host proteins. Glucosamine (UDP-N-acetyl)-2-epimerase/N-acetylmannosamine kinase (GNE) was identified as an important host factor in influenza A virus infection by genome-wide CRISPR screening. GNE knockout (KO) extensively inhibited the replication of multiple subtype influenza A viruses. It indirectly participated in IAV adsorption and endocytosis by regulating the expression of sialic acid (Sia), the main receptor of IAV. This finding provides novel insights into the replication mechanism of IAV.

## INTRODUCTION

Influenza A virus (IAV) primarily infects the epithelium of the upper respiratory tract (URT), trachea, and bronchi. Due to the lack of proofreading mechanism, the virus has a high mutation rate during replication (1 – 3). In addition, vaccination and the use of antiviral drugs, such as amantadine, can lead to the emergence of resistant strains (4 – 6). Therefore, the key host factors required for the influenza A virus infection are novel targets for antiviral drug development (7 – 9).

The influenza A virus life cycle highly depends on the host factors, including virus adsorption, endocytosis, transcription and replication, virus budding, and more (10). Genome-wide RNA interference (RNAi) screening and proteomic analysis have previously identified numerous host factors involved in influenza A virus replication (11 – 16). Recently, a GeCKO (genome-wide CRISPR knockout) screening strategy based on CRISPR/Cas9 gene editing technology has identified various host factors related to viral replication during infections such as human immunodeficiency virus (HIV), West Nile virus (WNV), Zika virus (ZIKV), severe acute respiratory syndrome coronavirus 2 (SARS-CoV-2), and influenza virus (17 – 23). For instance, Han et al. reported that SLC35A1 is crucial for sialic acid (Sia) synthesis and H5N1 adsorption and identified CIC as a negative regulator of cellular intrinsic immunity (18). Li et al. identified CMTR1 as necessary for effective influenza virus cap capture and regulation of the cellular autonomic immune response (23). Yi et al. revealed that CYTH2 mediates endosomal transport in the early stages of influenza virus infection (24). Zhou et al. reported that COG8 is essential for the retrograde transport of M2 from the endosome to the trans-Golgi network (25).

In this study, to identify the host factors required for influenza A virus replication, a genome-wide CRISPR/Cas9 screening was conducted in human lung epithelial cells (A549 cell lines). The results showed that glucosamine (UDP-N-acetyl)-2-epimerase/N-acetylmannosamine kinase (GNE) plays a critical role in IAV replication by regulating Sia synthesis. The GNE is involved in the sialylation of glycoproteins and glycolipids, which play important roles in biological processes such as cell communication, cell migration, and protein function. GNE mutation has been linked to saliuria, autosomal recessive inclusion body myopathy, and wild myopathy (26, 27). Furthermore, GNE knockout mice fail to survive during embryonic development (28). Differential sialylation of cell surface molecules is also associated with tumorigenicity and metastatic behavior of malignant cells. However, the role of the GNE in virus replication remains largely unclear.

GNE is a bifunctional enzyme that contains an N-terminal epimerase domain and a C-terminal kinase domain (29). It acts as a rate-limiting enzyme in the sialic acid biosynthesis pathway by regulating the biosynthesis of N-acetylneuraminic acid (Neu5Ac), which is the precursor of virtually all naturally occurring sialic acids (30, 31). In mammals, Neu5Ac and its activated nucleotide sugar CMP-Neu5Ac are synthesized in the cytosol from UDP-N-acetylglucosamine (UDP-GlcNAc) through five consecutive reactions, and GNE catalyzes the first two steps in this biosynthesis process (32 – 34). Influenza A virus attaches to cells by binding to sialic acid, which is expressed at the end of the cell surface glycoprotein and carbohydrate-lipid carbohydrate chains (35). Therefore, knockout GNE inhibited the adsorption efficiency of the influenza A virus on the cell surface by downregulating sialic acid biosynthesis, which suggested that the GNE is a potential target for anti-influenza drug development.

## RESULTS

### Establishment and screening of genome-wide CRISPR/Cas9 knockout library in A549 cells

**Fig 1:**
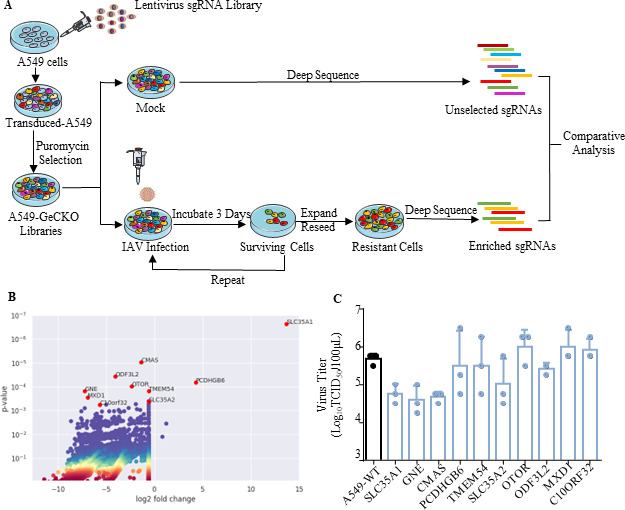
Genome-wide CRISPR/Cas9 screening for host factors involved in IAV infection. (**A**) Schematic of the generation of the A549-GeCKO libraries and screening host factors associated with IAV infection. (**B**) The volcano plot shows the enriched genes in the screening, and the top 10 genes are highlighted in red dots. The vertical axis represents the confidence *P* value, and the horizontal axis represents the enrichment amount of sgRNAs corresponding to the gene. (**C**) A/WSN/1933 H1N1 (WSN) virus replication titers in different gene-editing polyclonal cells. The gene-editing polyclonal cells and A549 WT cells were infected with the WSN virus [multiplicity of infection (MOI) = 0.1], and the supernatants were collected at 24 hours postinfection for viral titration in Madin-Darby canine kidney (MDCK) cells.

To identify host factors required for influenza A virus infection, a genome-wide CRISPR/Cas9 screening in A549 cells was conducted as illustrated in Fig. 1A. The Human CRISPR Knockout Pooled Library (GeCKO V2) contained, in total, 123,411 unique single-guide RNAs (sgRNAs) targeting 19,050 genes. The lentivirus library was packaged in 293T cells and infected A549 cells with a low dose of lentivirus. The A549 GeCKO cell libraries were obtained after 14 days of puromycin screening.

The pooled A549-GeCKO cell libraries were infected with A/WSN/1933 H1N1 (WSN) at a multiplicity of infection (MOI) of 5. Surviving cells were reseeded for three rounds of virus infection. Genomic DNA was extracted from the surviving cells, and integrated sgRNAs were amplified by PCR and sequenced. We repeated the screening three times with different strains of influenza A virus; 10 host genes with the highest enrichment were identified in three independent experiments (Fig. 1B). To further explore the role of the 10 genes in influenza virus replication, further single gene editing results showed that editing SLC35A1, MX1, CMAS, and GNE significantly inhibited the influenza virus replication. SLC35A1, MX1, and CMAS have previously been identified by others as host genes associated with influenza A virus replication (Fig. 1C).

### Generation of GNE knockout cell lines

**Fig 2:**
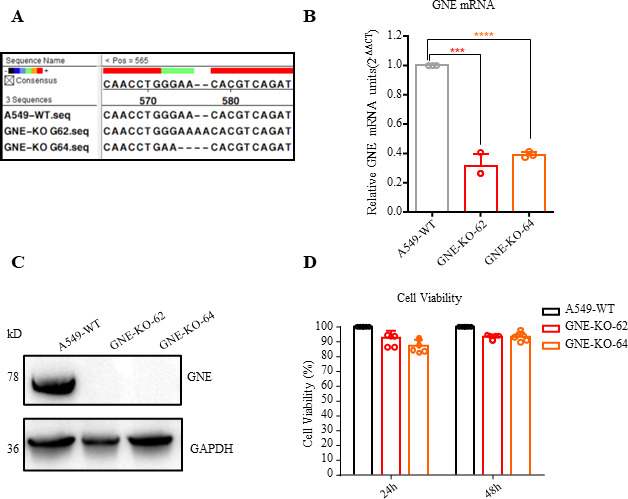
Construction and verification of GNE knockout cell lines. (**A**) Gene editing in GNE-KO-G62 and GNE-KO-G64 cells was verified by sequencing. The genomic DNA of cells was extracted to amplify GNE gene fragments, and then Sanger sequencing was performed. (**B**) mRNA transcription levels in GNE knockout cells were verified by RT-qPCR, GAPDH was used as an internal reference, and relative expression levels were calculated by 2^-△△CT^. (**C**) The protein expression level in GNE knockout cells was verified by Western blotting and compared with that of the A549 WT cells. (**D**) The viability of GNE-KO cells and A549 WT cells was measured by CCK-8 assay at 24 hours and 48 hours, respectively. The data shown are from five biological replicates (means ± SDs).

To investigate the function of GNE, two GNE-KO A549 cell lines were generated by CRISPR-Cas9 technology. Sanger sequencing confirmed the successful transduction of GNE-specific sgRNA in GNE-KO G62 and GNE-KO G64, which resulted in the 7-bp deletion and 2-bp insertion in the ORFs of GNE, respectively (Fig. 2A). The insertion/deletion led to premature termination of GNE protein translation. The knockout efficiency was verified by Western blot and reverse transcription-quantitative PCR (RT-qPCR), which showed that GNE had been knocked out in both GNE-KO-G62 and GNE-KO-G64 (Fig. 2B and C). Importantly, the cell viability of these GNE-KO cells was not significantly different from that of wild-type A549 cells at both 24 and 48 hours (Fig. 2D).

### GNE is involved in IAV replication

**Fig 3:**
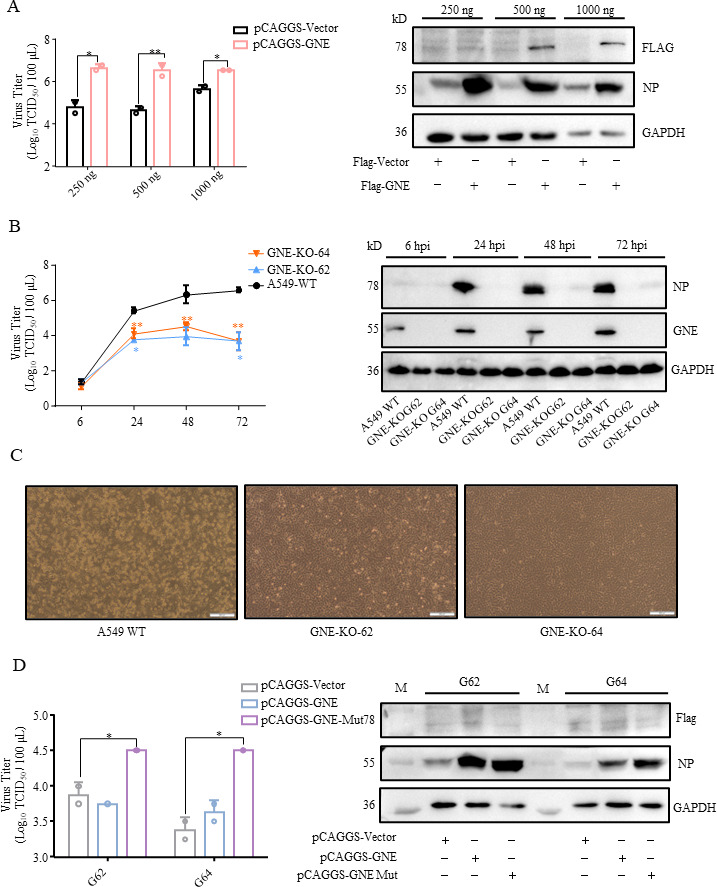
GNE is involved in influenza A virus infection. (**A**) Overexpression of GNE promotes the replication of WSN. The A549 cells were transfected with different doses of pCAGGS-Vector and pCAGGS-GNE (250 ng, 500 ng, and 1000 ng). WSN was inoculated 24 hours after transfection (MOI = 0.1), and the supernatant and cells were collected 48 hours postinfection. The supernatant was titrated in MDCK cells, and the cells were collected to detect the NP level by Western blotting. (**B**) Replication of WSN virus in GNE-KO and A549 WT cells. GNE-KO and A549 WT cells were infected with WSN (MOI = 0.001), the supernatants were collected at the indicated time points for virus titration in MDCK cells, and the cells were collected to detect the NP level by Western blotting. (**C**) Observation of cytopathic effects of GNE-KO and A549 WT cells at 3 days postinfection. (**D**) pCAGGS-Vector, pCAGGS-GNE, and pCAGGS-GNE-Mut were overexpressed in GNE-KO-G62 and GNE-KO-G64 cells. WSN virus was inoculated 24 hours after transfection (MOI = 0.1), the supernatants were collected 48 hours postinfection to determine titer, and the cells were collected to detect the NP level by Western blotting.

To investigate the role of GNE in IAV replication, overexpression of GNE increased the replication of WSN to about 10-fold compared to the mock group (Fig. 3A). The growth curves of the virus in wild-type cells and GNE-KO cells showed that GNE knockout significantly inhibited the replication of A/WSN/1933 H1N1 (WSN) (Fig. 3B). Moreover, the influenza A virus caused serious cytopathic effect (CPE) in WT A549 cells, but not in the GNE-KO-G62 and GNE-KO-G64 cells (Fig. 3C). Indirect immunofluorescence assay (IFA) also showed that the proportion of infected cells [hemagglutinin (HA) and nucleoprotein (NP) fluorescence positive] of GNE-KO-G62 and GNE-KO-G64 was significantly lower than that of WT A549 cells (Fig. S1).

To further confirm the role of GNE in influenza A virus replication, the complementation experiments were conducted by introducing exogenous native GNE and GNE-Mut (synonymous mutation in the sgRNA region) expression plasmids in two KO cells. The GNE-Mut expression plasmid increased the replication level of WSN in two KO cells, whereas the complement of exogenous native GNE expression plasmid did not improve the replication level of WSN (Fig. 3D), indicating that the GNE-sgRNA interfered with the native GNE expression and thus resulted in the inhibition of WSN replication. These results confirmed that GNE is indeed a host factor required in WSN replication.

### GNE plays an important role in multiple subtype influenza viruses and Newcastle disease virus (NDV) replication

**Fig 4:**
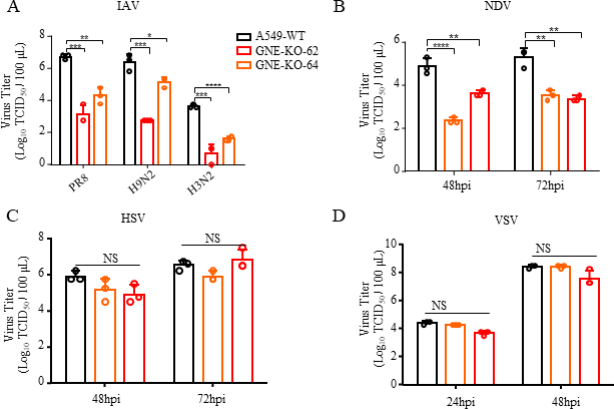
Effect of GNE knockout on different virus replication. (**A**) Replication titers of different influenza A virus strains in A549 WT cells and GNE-KO cells. A549 cells and GNE-KO cells were infected with the indicated IAV strains (MOI = 0.001), and the supernatants were harvested at 48 hours postinfection for virus titration in MDCK cells. (**B–D**) Replication titers of different viruses in A549 WT cells and GNE-KO cells. GNE-KO cells and A549 WT cells were infected with NDV, herpes simplex virus (HSV), and vesicular stomatitis virus (VSV) (MOI = 0.001), respectively, and the supernatant was collected for viral titration. The data shown are from three biological replicates (means ± SDs).

To determine whether GNE was crucial for replication of different subtype influenza A viruses, the GNE-KO and WT A549 cells were infected with A/Puerto Rico/8-SV14/1934(PR8/H1N1), A/swine/Guangxi/2518/2011(2518 /H3N2) and A/chicken/Jiangsu/A2093/2011 (A2093/H9N2). The results showed that GNE-KO significantly inhibited the replication of H1N1, H3N2, and H9N2 influenza A virus strains (Fig. 4A).

To further explore the role of GNE in the replication of other viruses, the replication of VSV (single-negative-stranded RNA virus), NDV (single-negative-stranded RNA virus), and HSV (double-stranded DNA virus) was tested in the GNE-KO and WT A549 cells. The results showed that only NDV replication was significantly inhibited in GNE-KO cells, while GNE knockout did not influence in the replication of other viruses (Fig. 4B through D).

### GNE-KO affects sialic acid receptor synthesis

**Fig 5:**
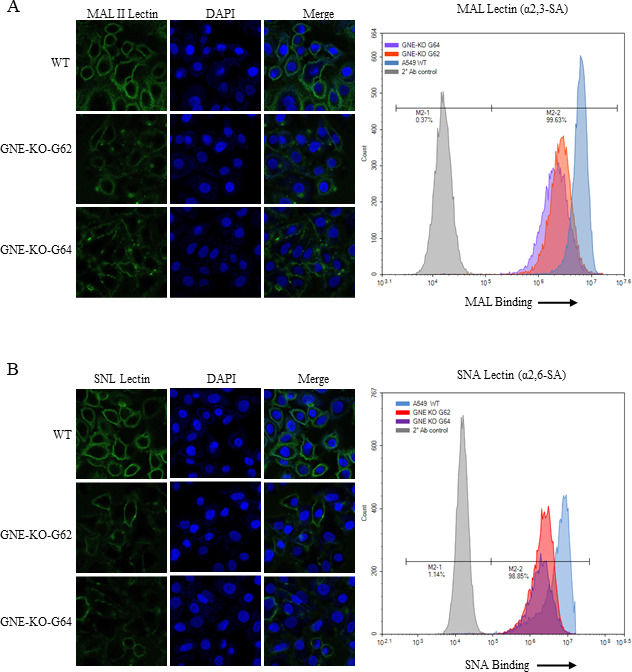
α-2,3- and α-2,6-linked-sialic acid receptor expression in the cell surface. A549 WT cells and GNE-KO cells were fixed with 4% formaldehyde for 10 minutes, washed with phosphate-buffered saline (PBS) twice, and incubated with 20-µg/mL biotinylated. (**A**) *Maackia amurensis* lectin II or (**B**) *Sambucus nigra* lectin for 1 hour on ice, followed by staining with 1-µg/mL Alexa Fluor 488-conjugated streptavidin, and then observed with a confocal microscope or analyzed by a flow cytometer.

Given the role of GNE in sialic acid biosynthesis pathway, the expression of different types of sialic acid on the surface of GNE-KO cell lines were tested by using biotinylated *Maackia amurensis* lectin (MAL II) and *Sambucus nigra* lectin (SNL), which specifically bind to α-2,3-linked-sialic acid receptor and α-2,6-linked-sialic acid receptor on host cells, respectively. GNE-KO-G62, GNE-KO-G64, and A549 WT cells were incubated with biotinylated MAL II or SNL, and the biotin-streptavidin system was used to detect the distribution and amount of sialic acid on the cell surface by confocal microscope and flow cytometry. The results showed a significant decrease in the amount of sialic acid detected by both types of lectins on the cell surface of GNE-KO cells compared to WT cells, indicating a loss of cell-surface sialic acid in the GNE-KO cells (Fig. 5A and B). These findings suggest that the GNE is involved in the synthesis or maintenance of sialic acid on the cell surface, which may explain why replication of both influenza A virus and NDV was significantly inhibited in GNE-KO cells, as both viruses bind to sialic acid receptors.

### GNE is essential for influenza A virus adsorption and endocytosis

**Fig 6:**
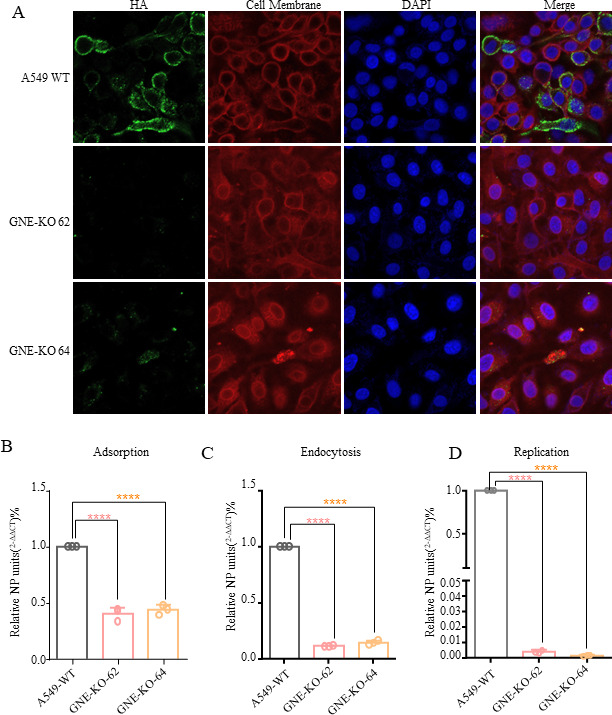
GNE knockout affects viral adsorption, endocytosis, and replication. (**A**) HA binding assay. The A549 WT cells and the GNE-KO cells were infected with IAV for 1 hour on ice (MOI = 3), and then the HA protein was detected by indirect immunofluorescence and observed under a confocal laser microscope. (**B–D**) GNE-KO and A549 cells were infected with WSN (MOI = 3), and the cell samples of A549 WT and GNE-KO cells were absorbed on ice for 1 hour, endocytosis for 1 hour, and replication for 6 hours. RNA was extracted to detect the copy number of the virus genome. The data shown are from three biological replicates (means ± SDs).

To further investigate whether the GNE knockout impedes IAV adsorption, the GNE-KO and WT cells were infected with WSN strain on ice for 1 hour (MOI = 5), allowing attachment but not internalization. After fixing the cells with 4% paraformaldehyde, the HA proteins were stained by a specific antibody. The result showed a significant reduction in viral attachment in GNE-KO cells compared to WT cells, as GNE-KO cells had significantly lower levels of virions on their cell surface (Fig. 6A).

To further confirm this result, the virus load was also detected at three stages of viral replication: adsorption, endocytosis, and replication. The results showed that GNE-KO significantly affected the adsorption of IAV, as the amount of virus adsorbed on the cell surface was about one-third of that in WT cells (Fig. 6B). Furthermore, the endocytosis efficiency also decreased after GNE knockout. In order to accurately quantify the virions inside the cells, neuraminidase is used to cut the connection between the virions and the receptor to remove the interference of the virions on the cell surface. The results showed that the endocytosis of virions in GNE-KO cells was one-tenth of that of WT cells (Fig. 6C). This suggests that GNE also plays a role in the endocytosis of IAV. The detection of viral replication 6 hours postinfection showed that the virus load in GNE-KO cells is 1,000 times lower than that in A549 WT cells (Fig. 6D).

### Innate immune response was enhanced in GNE-KO cells

**Fig 7:**
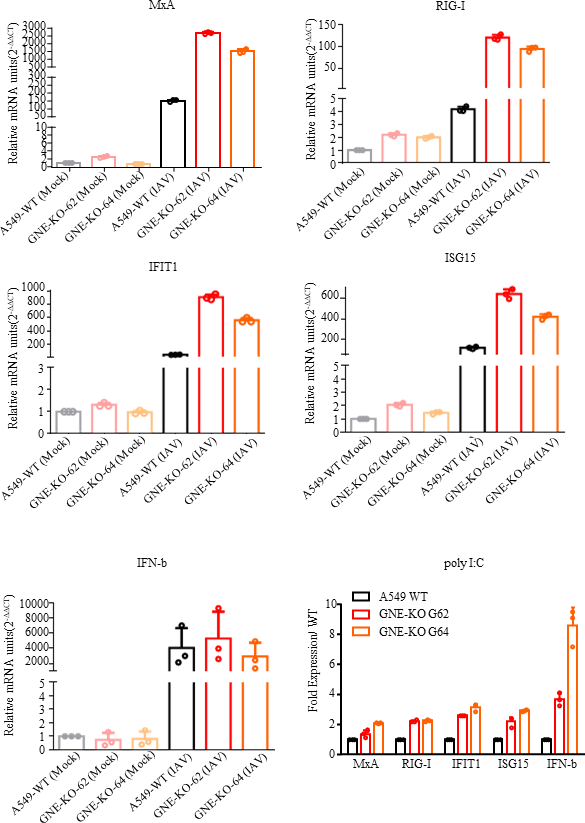
The innate immune response of GNE-KO cells and A549 cells upon influenza virus A infection or poly(I:C) stimulation. A549 WT cells and GNE-KO cells were stimulated with IAV or poly(I:C), and cell samples were collected 24 hours postinfection for RT-qPCR to detect transcription levels of innate immune-related genes: MxA, RIG-I, ISG15, IFIT1, and IFN-B. The data shown are from three biological replicates (means ± SDs).

Innate immunity is the first line of defense against virus invasion. The innate immunity response of the GNE-KO cells was evaluated, and the results showed that interferon-related innate immunity genes (MxA, RIG-I, ISG15, IFIT1) in GNE-KO cells were upregulated significantly higher than those in WT cells infected with influenza A virus or stimulated by polyinosinic-polycytidylic acid [poly(I:C)]. Furthermore, the interferon-β expression in GNE-KO cells was significantly upregulated in response to poly(I:C) stimulation (Fig. 7). The strong innate immune response in GNE-KO cells also inhibited viral replication, while the underlying mechanism of stronger innate immunity response observed in GNE-KO cells remains unknown.

## DISCUSSION

The GNE was identified as a key host factor required for influenza A virus infection. The GNE protein is a bi-functional enzyme; the amino-terminal domain, consisting of 1–400 amino acids, is mainly responsible for epimerase activity that converts UDP-GlcNAc to N-acetylmannosamine (ManNAc). The carboxy-terminal domain, consisting of 400–722 amino acids, is mainly responsible for kinase activity (36), which converts mannose to mannose 6-phosphate through phosphorylation using ATP. In this study, the sgRNA for GNE is located at the 350 amino acid position. Gene editing at this position disables the expression of the kinase domain. The function of this kinase domain in sialic acid synthesis is unknown (37, 38). However, our gene deletion study confirmed that the kinase domain responsible for phosphorylation is essential for sialic acid synthesis.

There are three different isoforms of human GNE, namely, hGNE1, hGNE2, and hGNE3. GNE1 was generally expressed, while GNE2 and GNE3 show tissue-specific expression patterns (39, 40). The three GNE isoforms differ only in the N-terminus, with hGNE2 possessing an extended 31 additional amino acids in the N-terminus and hGNE3 having 55-amino acid deletion replaced by 14 new amino acids in the N-terminus. Studies have speculated that GNE1 may be primarily responsible for the expression of sialic acid, while GNE2 and GNE3 involved in the subtle regulation (35, 41, 42). Although the exact functions of these three isoforms are not yet clear, the gene editing in the GNE-KO cell is generated at the 350 amino acids, which allows for interference with all three isoforms and completely blocks the three GNE isoforms expression. In the complementation experiments, we only introduced hGNE1 cDNA into GNE-KO cells and observed a significant recovery of the influenza A virus replication. This further highlights the crucial role of the GNE1 isoform in the expression of sialic acid.

In our study, we confirmed that GNE is not a pan-host factor that affects the replication of all viruses but is involved in the infection of sialic acid-receptor viruses, such as influenza A viruses and Newcastle disease viruses. Among the several enzymes involved in sialic acid biosynthesis, only NANP is dispensable, whereas GNE, NANS, and CMAS are essential for Sia synthesis (34). It has been reported that CMAS knockout affects sialic acid synthesis and thus interferes with influenza A virus replication in newborn pig trachea epithelial (NPTr) cells (43), Our study elucidated that GNE is essential for influenza A virus replication in A549 cells.

Innate immunity is the first line of defense against virus invasion. The results showed that the IFN expression levels were higher in GNE-KO cells upon influenza A virus infection or poly(I:C) stimulation. It is widely known that IAV infection must evade innate immune defenses to successfully replicate, and GNE may be directly or indirectly involved in this process. The heightened interferon response observed in GNE-KO cells further restricted IAV replication. While the mechanism of heightened interferon response in GNE-KO cells remains unknown, it is possible that GNE is critical for the sialylation of some endogenous negative regulators of the IFN pathway. GNE-KO inhibited the sialylation and function of the negative regulator and then led to enhanced IFN response. Further research is needed to investigate the role of GNE in the innate immunity. Overall, the GNE is an important host factor involved in influenza A virus replication, playing an essential role in multiple stages including viral adsorption, endocytosis, and inhibition of innate immunity.

## MATERIALS AND METHODS

### Cell culture and viruses

Human embryonic kidney cells (HEK293T cells), human lung carcinoma cells (A549 cells), chick embryo fibroblast cells (CEF cells), and MDCK cells were cultured in DMEM (Gibco, Cat.no. C11995500BT) supplemented with 10% fetal bovine serum (HyClone, UT), L- glutamine, and antibiotics. All cells were maintained with 5% CO2, 37°C. CEF cells were prepared from 9-day-old specific pathogen-free (SPF) embryos (Boehringer Ingelheim) as previously described (44).

All influenza A virus strains used in the experiments, such as A/WSN/1933 H1N1 (WSN), A/PuertoRico/8-SV14/1934 (PR8/H1N1), A/Chicken/Shanghai/2093/2009 (A2093/H9N2), and A/swine/Guangxi/2518/2011 (GX2518, H3N2), were propagated in 9-day-old specific pathogen-free eggs and tittered on MDCK cells. NDV-Lasota strain was propagated in 9-day-old SPF eggs and tittered on CEF cells. HSV and VSV were propagated and tittered on A549 cells. The viruses used in this paper were preserved in the Laboratory of Influenza Pathogenic Ecology, Shanghai Veterinary Research Institute.

### A549-GeCKO library generation and host factor screening

Human CRISPR Knockout Pooled Library (GeCKO V2) was purchased from Addgene (catalog no.1000000048). psPAX2 and PMD2.G used for lentivirus packaging were preserved in our laboratory. The pooled library plasmids were transformed into Stbl3 competent cells by electroporation and extracted in large quantities. Then, the library plasmids were co-transfected with psPAX2 and PMD2.G into 293T cells to package library lentivirus, the supernatant was harvested at 48 hours after transfection, and A549 cells were infected with lentivirus (MOI = 0.3). The A549 GeCKO library cells were obtained by continuous pressure screening with 1-µg/mL puromycin for 14 days to kill the cells that were not infected with lentivirus.

A549-GeCKO library cells were infected with IAV at an MOI of 5, 3 days postinfection. The surviving cells were harvested and reseeded for three rounds of infection, and genomic DNA was isolated from these surviving cells by QIAamp DNA Mini Kit (catalog no. 51304, Qiagen, Germany) per the manufacturer’s specifications. The integrated sgRNA cassette was PCR amplified using Phanta Max Super-Fidelity DNA Polymerase (catalog no. P505-d3; Vazyme, Nanjing, China) and then submitted for high-throughput sequencing. PCR fragments were assessed with an Agilent 2100 Bioanalyzer and sequenced on an Illumina HiSeq2000 platform (GENEWIZ Biotechnology Co., LTD, Suzhou, China).

### Generation of the GNE knockout cells

The GNE-targeting sgRNA (5′-TTCCAATCTGACGTGTTCCC-3′) was designed according to the dedicated website (https://zlab.bio/guide-design-resources). Gene knockout was performed by cloning sgRNA into the LentiCRISPR V2 vector (Addgene, 52961) via *BsmBI* restriction sites. The lentivirus was packaged in HEK293T cells and used to infect WT A549 cells. Polyclonal GNE-KO cells were obtained by puromycin screening in A549 cells infected with packaged GNE-specific lentivirus, and GNE-KO monoclonal cells were obtained by limited dilution in 96-well plates. The knockout of GNE was confirmed through gDNA sequencing and western blotting.

### Cell viability assay

Cell viability was determined by using the Cell Counting Kit-8 (catalog no. CK04; Dojindo, Japan). Briefly, WT cells and GNE-KO cells were seeded in 96-well plates and cultured in a 37°C incubator, and 10 µL of CCK-8 reagent was added to each well at 24 hours and 48 hours and then incubated for another 1 hour. Cell viability was determined by measuring the absorbance at 450 nM in a universal microplate reader.

### Growth curve

To evaluate the growth of different viruses after GNE knockout, the A549 WT cells and GNE-KO cells were seeded in triplicate at a density of 3 × 10^5^ cells per well into 12-well plates, and cell numbers were measured the next day prior to infection. For IAV infection, cells were washed twice with phosphate-buffered saline (PBS), and OptiPRO SFM (catalog no.12309019; Gbico) with 0.2-µg/mL TPCK was used as infection medium and inoculated with IAV (different MOI was used for different infection experiments). After incubation for 1 hour at 37°C, the inoculum was removed, and cells were washed twice with PBS and placed in 1 mL of fresh infection medium. The supernatant was collected for viral titration at different time points. For NDV, HSV, and VSV infection, the infection medium was replaced with DMEM containing 2% fetal bovine serum, otherwise identical to IAV.

### Virus binding and endocytosis assay

To test the IAV binding to the cell surface, WT cells and GNE-KO cells were infected with IAV at the MOI of 3 in the infection medium and incubated on ice for 1 hour. The cells were washed with cold PBS (on ice) twice to remove unbound virus, and cell lysates were harvested. The amount of viral RNA was determined by RT-qPCR.

To test virus endocytosis in the infected cells, WT cells and GNE-KO cells were infected with IAV at the MOI of 3 in the infection medium and incubated on ice for 1 hour. The cells were incubated with pre-warmed infection medium for 0.5 hours at 37°C followed by 0.1-U/mL neuraminidase treatment for 30 minutes and then washed twice with PBS to remove the attached but not yet internalized virions, and cell lysates were harvested. Total cellular RNA was extracted and quantified by RT-qPCR.

To test the virus one round replication in cells, the WT cells and GNE-KO cells were infected with IAV at the MOI of 3, the cells were incubated at 37°C for 6 hours (a round of replication was completed) and then washed twice with PBS to remove the virions in the supernatant, and cell lysates were harvested. Total cellular RNA was extracted and quantified by RT-qPCR.

### Sialic acid detection on the cell surface

Biotinylated *Sambucus nigra* lectin and *Maackia amurensis* lectin II (catalog no. B-1305–2 and B-1265–1, respectively; Vector Lab, Burlingame, CA, USA) were used to detect the distribution of sialic acid receptors on the cell surface. Briefly, WT cells and GNE-KO cells were fixed with 4% formaldehyde for 10 minutes, washed with PBS twice, and incubated with 20-µg/mL biotinylated lectin for 1 hour on ice, followed by staining with 1-µg/mL Alexa Fluor 488-conjugated streptavidin (catalog no. 35103ES60; YEASEN, Shanghai, China). The sample nuclei were stained with DAPI for 10 minutes and then observed with a confocal microscope (LSM 880; Zeiss, Oberkochen, Germany) or analyzed by a flow cytometer (ACEA NovoCyte).

### Indirect immunofluorescence assay

The WT cells and the GNE-KO cells were infected with IAV for 1 hour on ice (MOI = 3), which allowed attachment but prevented internalization. Then, the cells were fixed with 4% formaldehyde for 10 minutes and blocked with 5% (wt/vol) skim milk for 30 minutes. After that, the cells were incubated with anti-HA antibodies at 4°C overnight and incubated with an appropriate fluorescent secondary antibody for 1 hour. The nuclei were stained with DAPI for 10 minutes at room temperature. These samples were observed and imaged with a Zeiss confocal microscope (LSM880, Germany).

### Real-time quantitative PCR analysis

RNA was extracted using an RNAsimple total RNA kit (Tiangen) according to the manufacturer’s instructions. The reverse transcription of the RNA sample was carried out by HiScript II Q Select RT SuperMix for qPCR (+gDNA wiper) (Vazyme, Nanjing, China), mRNA reverse transcription was performed using oligo(dT), and IAV vRNA reverse transcription was performed using Uni12(5′-AGCAAAAGCAGG-3′). SYBR green-based qPCR analysis of the resulting complementary DNA (cDNA) was performed using the ChamQ Universal SYBR qPCR Master Mix (Vazyme, Nanjing, China) on an Applied Biosystems QuantStudio 5 Real-Time PCR System. Primer sequences that were used for the qPCR are presented in Table 1. The gene expression values were calculated according to the 2^-ΔΔCT^ method, and the results are presented as the log^2^ fold change.

**TABLE 1:** Primer sequences that were used for the RT-qPCR

Gene name	Forward primer (5′−3′)	Reverse primer (5′−3′)
RIG-I	CCTACCTACATCCTGAGCTACAT	TCTAGGGCATCCAAAAAGCCA
IFIT1	AGTGTGGGAATACACAACCTACT	GGTCACCAGACTCCTCACATTT
ISG15	TCCTGGTGAGGAATAACAAGGG	GTCAGCCAGAACAGGTCGTC
MxA	GAAGGGCAACTCCTGACAGT	GTTTCCGAAGTGGACATCGCA
IFN-b	TCTGGCACAACAGGTAGTAGGC	GAGAAGCACAACAGGAGAGCAA
NP	GCCAGAATGCCACTGAAATC	AATCAGCCGTCCCTCATAATC
GAPDH	GGAGCGAGATCCCTCCAAAAT	GGCTGTTGTCATACTTCTCATGG
GNE	CCTGTGTGGGTAGACAATGATG	CGATTCCTGTGCCTGTGATAAG

### Western blotting

Cell extracts were lysed using RIPA lysis buffer (catalog no. P0013D; Beyotime, Shanghai, China) containing protease inhibitor PMSF, mixed with the sample containing 5 × SDS loading buffer, and denatured at 95°C for 10 minutes. Protein samples were resolved by SDS-PAGE, transferred to NC membranes (catalog no. 66485, PALL), blocked with 5% skim milk at room temperature for 1 hour, and incubated with the corresponding primary and secondary antibodies in turn. Antibodies used for western blot analysis are as follows: anti-GNE rabbit monoclonal antibodies (catalog no. ab189927; Abcam, Cambridge, UK); anti-GAPDH mouse monoclonal antibodies (catalog no. 60004–1-Ig; Proteintech, Wuhan, China); anti-FLAG-tag monoclonal antibodies (catalog no. M185-3L; MBL, Beijing, China); anti-IAV NP, M1, and HA rabbit polyclonal antibodies (catalog no. GTX125989, GTX125928, and GTX127357; GeneTex, Irvine, CA, USA); and Peroxidase-AffiniPure Goat Anti-Mouse IgG (H + L) and Peroxidase-AffiniPure Goat Anti-Rabbit IgG (H + L) ( catalog no. 115–035-003 and 111–035-003, respectively; Jackson, West Grove, PA, USA). Protein bands were visualized by LumiQ enhanced chemiluminescence substrate (catalog no.SB-WB012; Share-bio, Shanghai, China).

### Statistical analysis

Data were expressed as means ± standard deviation of three independent experiments. Statistical analysis was performed by determining *P* values using a paired two-tailed Student’s *t*-test (*, *P* < 0.05; **, *P* < 0.01; ***, *P* < 0.001, ****P < 0.0001).

## Data Availability

Sequences are available in the NCBI Sequence Read Archive under BioProject accession number PRJNA1012141.

## SUPPLEMENTAL MATERIAL

Fig. S1 spectrum.01643-23-s0001.pdf IFA results.

ASM does not own the copyrights to Supplemental Material that may be linked to, or accessed through, an article. The authors have granted ASM a non-exclusive, world-wide license to publish the Supplemental Material files. Please contact the corresponding author directly for reuse.
