# Supplementary material for: Genome-wide CRISPR screen identifies GNE as a key host factor that promotes influenza A virus adsorption and endocytosis
Source: Microbiol Spectr. 2023 Nov 15;11(6):e01643-23. doi: 10.1128/spectrum.01643-23 (PMC10715076; doi:10.1128/spectrum.01643-23)
Supplement: Fig. S1 — IFA results. [file spectrum.01643-23-s0001.pdf]

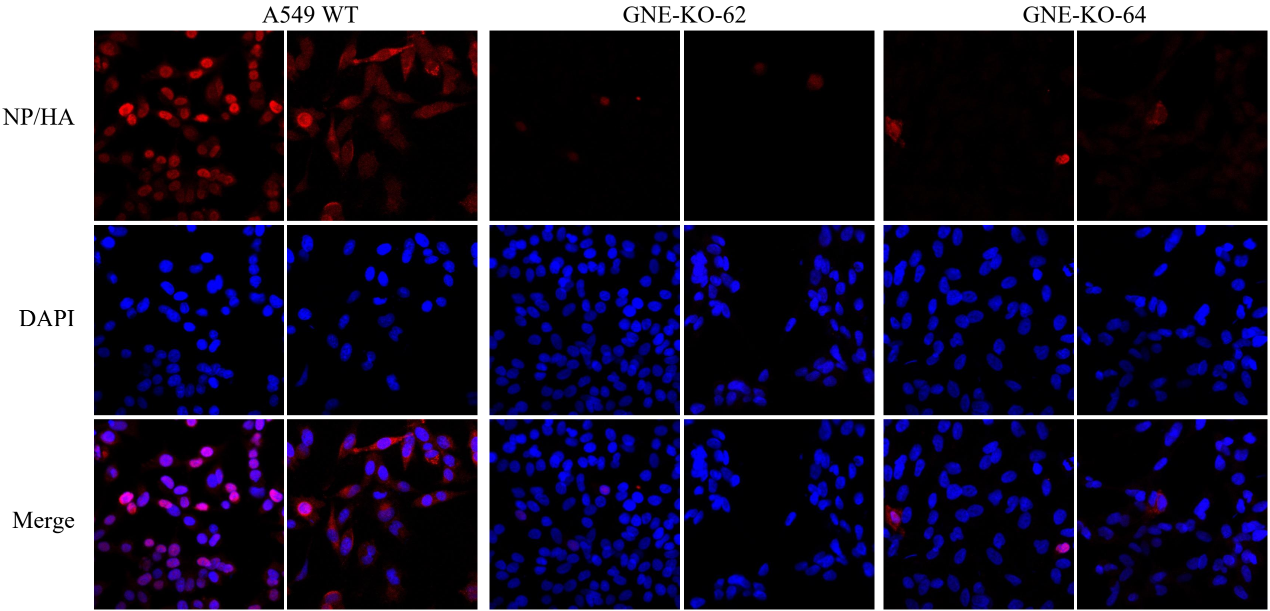

Figure S1. GNE knockout affects the efficiency of WSN infection. The expression levels of NP protein and HA protein were detected by indirect immunofluorescence 24 hours post infection with WSN in A549 WT cells and GNE-KO cells.
